# Case Report: Right Insular Stroke Causing Simultaneous Onset of a Functional Vestibular Disorder and Psychiatric Disorder—Persistent Postural–Perceptual Dizziness and Post–stroke Depression

**DOI:** 10.3389/fpsyt.2022.841072

**Published:** 2022-04-21

**Authors:** David C. Fipps, Jeffrey P. Staab, Nicholas D. Allen

**Affiliations:** ^1^Department of Psychiatry and Psychology, Mayo Clinic, Rochester, MN, United States; ^2^Department of Otorhinolaryngology–Head and Neck Surgery, Mayo Clinic, Rochester, MN, United States

**Keywords:** insula, persistent postural perceptual dizziness (PPPD), depression, stroke, functional disorder

## Abstract

**Introduction:**

Persistent postural–perceptual dizziness (PPPD) is a chronic functional vestibular disorder that can be precipitated by acquired brain injuries. Poststroke depression (PSD) is the most common psychiatric sequela of stroke, affecting 33% of stroke survivors. Pathophysiologic mechanisms of PPPD and PSD are not fully understood.

**Case Report:**

A 40-year-old woman developed new, debilitating chronic dizziness exacerbated by her own motion and exposure to visual motion stimuli plus prolonged depressive symptoms, both beginning within days after a localized right insular stroke. A collaborative evaluation by specialists in neurology, otorhinolaryngology, optometry, and psychiatry concluded that the insular stroke caused simultaneous onset of PPPD and PSD.

**Discussion:**

Prior case reports described short-lived vertigo following insular strokes, but no long-term vestibular symptoms without ongoing nystagmus or gait ataxia. In this case, chronic dizziness and motion sensitivity continued in the absence of focal neurologic deficits, invoking the possibility that changes in functioning of brain networks subserving spatial orientation persisted despite otherwise adequate recovery from the stroke, a mechanism previously proposed for PPPD. This case also reinforced prior work implicating pathways through the insula in PSD. Co-occurrence of PPPD and PSD offers insights into simultaneous functions of the insula in multiple networks in human brain.

## Introduction

Persistent postural–perceptual dizziness (PPPD) is a chronic functional vestibular disorder characterized by persistent swaying and rocking (nonspinning) vertigo and unsteadiness exacerbated by patients' own movements and exposure to motion-rich environments (1). It is precipitated by conditions that cause vertigo or dizziness or disrupt balance such as peripheral or central vestibular disorders, including stroke (2). Posterior circulation strokes are the most common cerebrovascular causes of acute vestibular symptoms (3), but 16 cases of acute-onset, mostly transient, vertigo have been reported following infarctions of vestibular cortical regions, which include the right retroinsula, parietal operculum, and adjacent areas of parietal and temporal cortices in humans (4, 5). Functional magnetic resonance imaging (fMRI) studies of patients with PPPD suggest that chronic symptoms may be caused by functional rather than structural changes (i.e., reduced activity and connectivity) in vestibular cortical regions, visual cortex, inferior frontal gyrus, and hippocampus (6).

Anxiety and depressive disorders are common in patients with PPPD, each with prevalence of ~25% (7). Preexisting anxiety disorders may be a risk factor for development of PPPD, but depression is typically a sequela (2).

Depression is the most common psychiatric complication of stroke, affecting 33% of stroke survivors (8). While the pathophysiology of poststroke depression (PSD) is not fully understood, infarcts affecting the prefrontal cortex, anterior cingulate gyrus, insula, central striatum, hippocampus, and amygdala have been implicated (9). This suggests that PSD may arise from strokes affecting key nodes in neural networks that regulate affective and neurovegetative functions. A network model of PSD has largely supplanted previous concepts that focused on lesions localized to specific brain regions (e.g., left frontal lobe) that were thought to have a particular propensity to cause PSD when infarcted (10).

Herein, we report the case of a patient who developed PPPD and PSD simultaneously after a right insular stroke without focal neurologic sequelae. The simultaneous onset of these functional vestibular and psychiatric disorders highlights the insula as a crucial node in brain networks that support the important human tasks of spatial orientation and mood regulation.

## Case Report

Ms. H. was a 40-year-old, right-handed woman with a medical history of chronic migraine with aura, dyslipidemia, obesity, nonalcoholic fatty liver disease, endometriosis, and gastroesophageal reflux disease. She had a psychiatric history of generalized anxiety disorder (GAD) and trichotillomania. She had no history of previous vestibular or depressive symptoms.

**Figure 1:**
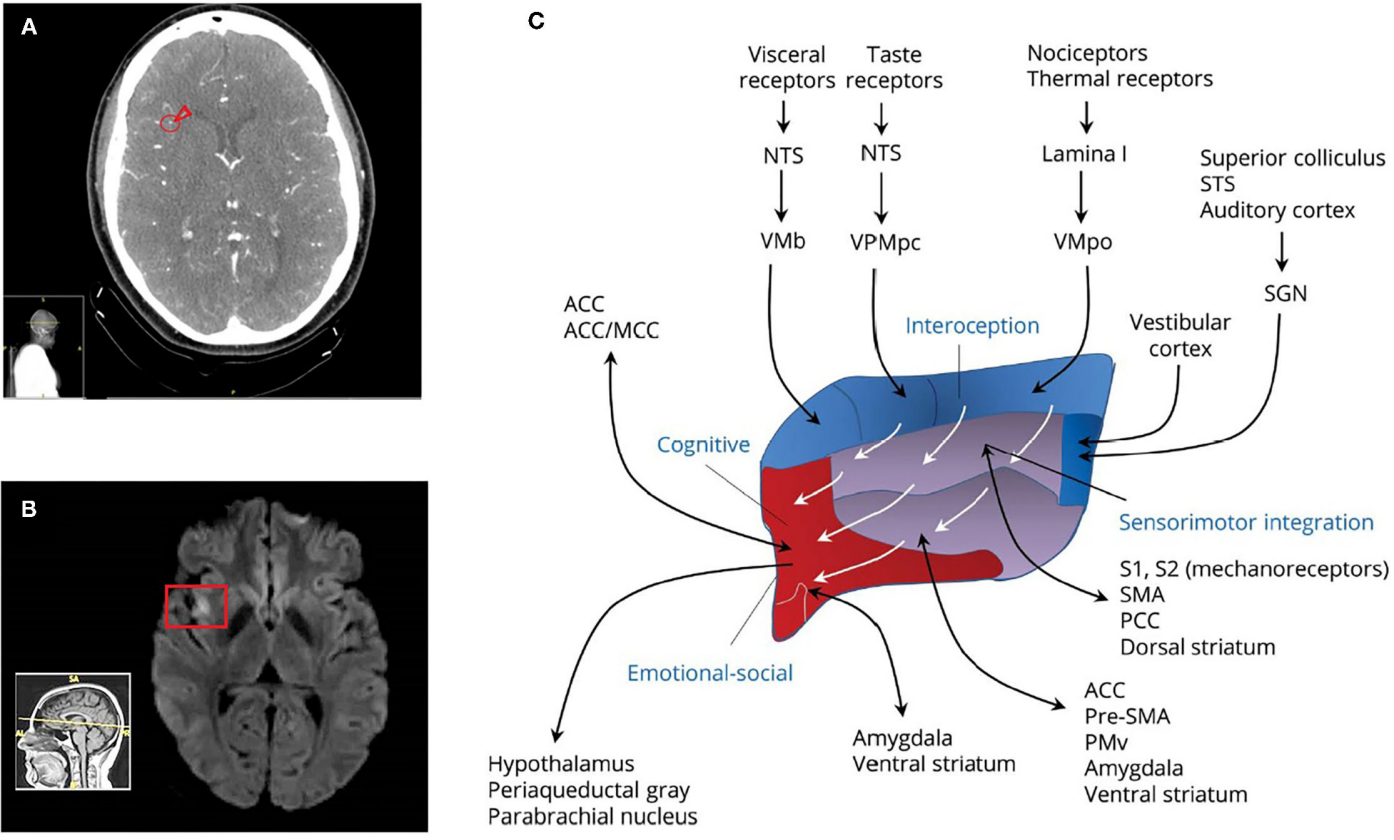
**(A)** Computed tomography angiography indicating short segment occlusion versus critical stenosis in the M2/M3 branch vessels of the rMCA. **(B)** Noncontrast MRI scan revealing a small evolving region of ischemia in the right insula adjacent to lesions in the CTA. **(C)** Complex interconnectivity of the insula with multiple circuits conveying interoceptive (blue area) sensorimotor (purple area), cognitive, emotional, and social functioning (red area). Reproduced and edited from Ghaziri et al. (11) with permission from Wolters Kluwer Health, Inc. ACC, anterior cingulate cortex; MCC, medial cingulate cortex; NTS, nucleus of the solitary tract; PPC, posterior parietal cortex; preSMA, presupplementary motor area; PMv, ventral premotor cortex; S1, somatosensory area 1; S2, somatosensory area 2; SGN, suprageniculate nucleus; SMA, Supplementary motor area; STS, superior temporal sulcus; VMb, ventromedial basal nucleus; VMpo, ventromedial posterior nucleus; VPMpc, ventral posteromedial nucleus.

Ms. H. presented to the emergency department at her local hospital after experiencing acute dysarthria and distal numbness in both arms. These symptoms had resolved by the time that she presented for evaluation. Medical records showed that her general physical examination was normal except for mild tachycardia. On neurologic examination, she was awake, alert, and fully oriented. Her pupils were equal, round, and reactive to light and accommodation. Visual fields were intact to confrontation. Examination of cranial nerves II–XII demonstrated no abnormalities, specifically no oculomotor deficits, including no spontaneous nystagmus. Motor strength was 5/5, sensation was intact to light touch in all extremities, and speech was normal. Finger-to-nose testing was performed normally. She also had a normal gait. National Institutes of Health Stroke Scale score was 0. Computed tomographic angiography (CTA) with and without contrast revealed stenosis within multiple M2/M3 branch vessels of the right middle cerebral artery (rMCA) (Figure 1A). Noncontrast MRI of the brain revealed a small region of restricted diffusion in the right mid insular cortex consistent with evolving ischemia (Figure 1B). She was admitted to her local hospital for acute treatment. She was outside the time window for tissue plasminogen activator, so she was treated emergently with aspirin 325 mg and clopidogrel 300 mg daily followed by heparin via continuous infusion due to concern for a partially occlusive thrombus. After 3 days on heparin, a repeat CTA showed reconstitution of blood flow within the M2/M3 segments of the rMCA. A transthoracic echocardiogram with bubble study found a large patent foramen ovale (PFO). Hypercoagulable panel and ultrasound of the legs were negative. She was discharged on aspirin 325 mg daily and rosuvastatin 40 mg daily. Two weeks later, she underwent closure of her PFO.

Within a few days of hospital discharge following her stroke, Ms. H. noticed gradual onset of vestibular and depressive symptoms that interfered with daily life. She described daily sensations of spinning vertigo, unsteadiness, and lightheadedness. These symptoms were exacerbated by her own movements such as standing upright or bending over quickly, by exposure to moving visual stimuli or complex visual patterns, and when performing tasks that required precise visual focus such as driving, watching television, or working on a computer. Spinning vertigo resolved gradually within days, but her other space and motion symptoms consolidated into a chronic pattern. They were exacerbated by subsequent attacks of migraine, but she did not develop a distinct pattern of vestibular migraine. There was no pattern of dizziness associated with cephalalgia, photophobia/phonophobia, nausea/vomiting, or aura. Ms. H. did not report symptoms suggesting bilateral vestibular loss (e.g., her visually induced symptoms occurred even when sitting still, and there was no head-motion–induced gait unsteadiness or oscillopsia).

Simultaneously, Ms. H. experienced onset of low mood and anhedonia with notable disturbances of sleep and appetite. Her daily activities were restricted by prominent fatigue. She developed burdensome feelings of guilt and worthlessness, but no hopelessness, helplessness, or suicidal ideation. She continued to have symptoms of preexisting GAD including excessive worry, restlessness, tension, difficulty concentrating, irritability, and sleep disturbance. Laboratory and radiologic evaluation completed at that time including complete blood count, comprehensive metabolic panel, thyroid-stimulating hormone, B_12_, and repeat head computed tomography demonstrated no new abnormalities.

Six months after her stroke, Ms. H. continued to report vestibular symptoms that interfered with activities such as riding in a car, being under fluorescent lights, watching traffic at busy intersections, going down escalators, and walking over patterned floors. She was referred to our tertiary center and underwent vestibular and oculomotor examinations that found normal smooth pursuit, saccadic, and convergence eye movements with no spontaneous or gaze holding nystagmus. Vestibulo-ocular reflex cancellation maneuvers were normal. Dix–Hallpike and roll tests were negative. Audiometric evaluation found normal hearing, acoustic reflexes, and Eustachian tube function.

Ms. H.'s psychiatric symptoms also continued with moderately severe depression on the Patient Health Questionnaire (score = 13) and mild anxiety on the Generalized Anxiety Disorder Scale (GAD-7; score = 7). Psychiatric consultation using the Mini International Neuropsychiatric Interview identified a first lifetime major depressive episode and confirmed the presence of preexisting GAD. There was no evidence of mania, hypomania, panic disorder, agoraphobia, posttraumatic stress disorder, psychosis, eating disorder, or illness anxiety.

## Discussion

The insula is a multifaceted neuronal structure with broad interconnectivity to other brain regions, including those involved in viscerosensory, visceromotor, somatosensory, somatomotor, motor association, and vestibular functions, language, and limbic integration (11). This case calls attention to its roles in two critical human activities, spatial orientation and mood regulation. In humans, vestibular cortical functions are centered in the middle, posterior, and retroinsular regions and parietal operculum, which constitute the analog of the parietoinsular vestibular cortex in other primates (12). Evidence from reports of unilateral infarctions in these regions (5, 13) and fMRI studies in normal individuals (14) showed that the right hemisphere dominates vestibular function in right-handed patients, as in this case. For spatial orientation, these areas are connected to multiple additional regions of the brain, including the hippocampi bilaterally (11).

Dieterich and Brandt (4) identified 10 cases of vestibular symptoms occurring after cortical strokes; 9 were in the insula. In a prospective study of 668 patients with stroke, Eguchi and colleagues (5) found eight cases of acute vertigo, including six with strokes in the insula or adjacent areas of temporal and parietal cortices. The others had infarcts of the hippocampus and putamen. In 13 of these 16 cases involving strokes of the insula or adjacent regions, vestibular symptoms and signs lasted only days (none >2 months), and half had benign neurologic examinations such as Ms. H. In the other three cases, persistent symptoms were accompanied by ongoing signs of vestibular dysfunction. Even among patients reporting acute vestibular symptoms or abnormal gait, typical signs of central vestibular pathology were not universal. For example, spontaneous and gaze-evoked nystagmus was absent in two of six patients with spinning vertigo and four of four patients with nonspinning vertigo or gait ataxia described by Dieterich and Brandt (4).

There are two potential precipitants of chronic vestibular symptoms in this case. One would be direct effects of structural lesions causing prolonged disruption of spatial orientation systems, and the other would be persistent functional changes within those same systems. In structural lesions, neurologic deficits localizing to the site of the lesion and dysfunction of interconnected networks such as those through the hippocampus would be expected. Considering that most reported insular strokes caused only transient vestibular symptoms and signs, however, other processes must be involved. In PPPD, acute disruption of vestibular and balance control is thought to trigger over reliance on visual inputs for spatial orientation and increased use of stiffened postural control for stance and gait that persist well beyond resolution of precipitants (2). These shifts in functioning may explain the sustained hypersensitivity to motion of self and surrounding objects without focal neurologic deficits. In addition, fMRI data suggest disruptions in hippocampal connectivity to vestibular (including insula) and oculomotor cortex and cerebellum (12), which could explain poor performance on spatial orientation tasks such as the Virtual Morris Water Maze Test in patients with PPPD (13). An anxiety diathesis such as Ms. H's GAD is a predisposing factor of PPPD (2).

Regarding PSD, structural and functional neuroimaging studies suggest that the insula is an important node in networks that regulate mood and emotional expression (14). Voxel-based morphometry found significant reductions in insular gray matter volume in patients with major depressive disorder (15, 16). Functional neuroimaging revealed insular activation to emotionally charged stimuli such as disgusting, scary, happy, sad, or sexual images (17).

Patients with chronic dizziness are prone to comorbid depression, although as a consequence rather than cause of vestibular symptoms (18). Unlike state and trait anxiety, there are no suspected brain mechanisms linking depression to onset of PPPD (2). Psychological factors, including catastrophic cognitive–emotional response to the stroke, may have played a role in the development of PSD and PPPD, as has been shown separately for both conditions (19). However, the simultaneous onset of PSD and PPPD in a patient who did not evidence that type of acute reaction suggests that the structural injury to the insula was a more direct precipitant. Therefore, in this case, it is likely that simultaneous development of PPPD and PSD arose from disruption of two separate neural networks, one subserving spatial orientation and the other mood regulation, passing through the insula. The neuroanatomical structure of the insula and its efferent and afferents connections to other brain regions (Figure 1C) suggests that the mid-insula location of Ms. H's stroke may have simultaneously disrupted the functioning of posterior pathways connected to vestibular function and sensorimotor integration and anterior ones affecting cognitive and emotional–social function.

## Data Availability Statement

The original contributions presented in the study are included in the article/supplementary material, further inquiries can be directed to the corresponding author.

## Ethics Statement

Ethical review and approval was not required for the study on human participants in accordance with the local legislation and institutional requirements. The patients/participants provided their written informed consent to participate in this study. Written informed consent was obtained from the individual(s) for the publication of any potentially identifiable images or data included in this article.

## Author Contributions

DF and NA clinically evaluated the patient. DF, NA, and JS performed the literature search. DF wrote the first draft, finalized the drafting, and submitted the manuscript. NA and JS edited and contributed revisions. All authors contributed to the article and approved the submitted version.

## Funding

JS was supported by the U. S. Department of Defense grant W81XWH1810760 via the Congressionally Directed Medical Research Program.

## Conflict of Interest

The authors declare that the research was conducted in the absence of any commercial or financial relationships that could be construed as a potential conflict of interest.

## Publisher's Note

All claims expressed in this article are solely those of the authors and do not necessarily represent those of their affiliated organizations, or those of the publisher, the editors and the reviewers. Any product that may be evaluated in this article, or claim that may be made by its manufacturer, is not guaranteed or endorsed by the publisher.
